# Unilateral Dilated Pupil and Spontaneous Cardiac Arrest with Successful Bystander Resuscitation

**DOI:** 10.1155/2017/4071531

**Published:** 2017-05-17

**Authors:** James M. Hancox, Julian Spiers, Nicholas Crombie, David N. Naumann

**Affiliations:** ^1^Midlands Air Ambulance, Hawthorn House, Dudley Rd, Stourbridge DY9 8BQ, UK; ^2^NIHR Surgical Reconstruction and Microbiology Research Centre, Queen Elizabeth Hospital, Birmingham B15 2TH, UK

## Abstract

A 75-year-old man collapsed on a golf course and received cardiopulmonary resuscitation from a bystander, including the use of a public automated external defibrillator (AED). The AED was discharged once, with return of spontaneous circulation. An air ambulance crew found the patient haemodynamically stable, with no acute abnormalities on a 12-lead ECG. He had reduced consciousness and a dilated left pupil. On contacting the patient's wife by telephone, she said that he had fallen and hit his head earlier that day. The crew decided to convey the patient to a Major Trauma Centre that had both neurosurgical and cardiology specialist services (rather than the nearest hospital) so that both traumatic brain injury and cardiac pathologies could be addressed if required. A head CT was normal, but coronary angiography demonstrated occlusion of two coronary arteries. These were successfully treated with stenting, and the patient went home two weeks later. He informed medical staff that his left pupil has been permanently dilated since he was a boy following a traumatic ocular injury. This case illustrates the utility of early deployment of an AED as well as the importance of an accurate history and emergency decision-making by prehospital personnel.

## 1. Introduction

Spontaneous collapse and cardiac arrest in a public place can be daunting for bystanders, but early deployment of a public access defibrillator may improve survival [1, 2]. Since collapsed patients are unable to give their own history, early assessment by emergency ambulance crews must depend on collateral (bystander and relative) history, as well as clinical signs. We present a case of a patient who had a spontaneous cardiac arrest at a rural golf club and was revived by the combination of bystander cardiopulmonary resuscitation and defibrillation. Early diagnosis was challenging due to a history of head trauma and a unilateral dilated pupil. Since both neurological and cardiological causes were plausible, the patient was conveyed to a centre with specialist expertise in both, rather than the nearest hospital. This case illustrates the requirement for early bystander resuscitation and careful decision-making by prehospital ambulance crew with regard to the most appropriate centre for evacuation of the patient due to the difficulty in determining the aetiology of cardiac arrest.

## 2. Case Presentation

An otherwise healthy 75-year-old man had a spontaneous collapse on a golf course, witnessed by other club members. He had no pulse and was not breathing and received cardiopulmonary resuscitation (CPR) from a bystander. After 15 minutes of CPR, a public automated external defibrillator (AED) was appropriately deployed, and he received one shock, following which he appeared to regain some consciousness.

Due to the rural location, the fastest prehospital transport to the scene was by air ambulance. A crew of two Critical Care Paramedics (CCPs) arrived to find the patient placed in the recovery position, spontaneously ventilating, with a palpable pulse, adequate blood pressure, normal oxygen saturation readings, and normal blood glucose. A 12-lead ECG showed no acute abnormalities.

The patient had a reduced Glasgow Coma Scale (GCS) and a unilateral fixed, dilated pupil. He exhibited trismus and occasional decorticate posturing. History from the patient's wife (via telephone) was that earlier in the morning he had slipped on some ice and fallen over, hitting his head, but had decided that he was well enough to play golf. Raised intracranial pressure was considered a possibility due to these signs. The patient was given high flow oxygen via a nonrebreathing mask and the airway was managed to good effect using basic manual techniques and a nasal pharyngeal airway.

Determining the aetiology of cardiac arrest in the prehospital environment can be difficult, and some of the differential diagnoses in this case included traumatic brain injury, acute myocardial infarction, or cerebrovascular accident. The CCPs made a decision to convey the patient to a Major Trauma Centre that had neurosurgical and cardiology specialist expertise, rather than the nearest hospital. When he arrived at the Emergency Department, he was anaesthetised and underwent rapid sequence induction. A head CT was performed, and this was reported as normal. He then proceeded to undergo coronary angiography, which demonstrated an occlusion of two coronary arteries. He underwent percutaneous coronary intervention (PCI) via the right radial artery, with angioplasty and stenting of the right coronary artery, left main stem, and proximal left anterior descending coronary artery.

**Figure 1:**
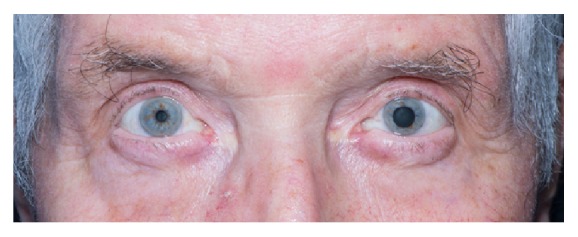
Dilated left pupil seen in comparison to contralateral side. This abnormality had been present since an ocular injury during a football game more than 45 years earlier. Informed consent was obtained for the use of this photo.

During his inpatient stay, he informed medical staff that his left pupil has been permanently dilated since he was a boy, following a traumatic ocular injury whilst playing football (shown in Figure 1).

## 3. Discussion

Although bystander CPR and use of public AEDs can be variable [3], they do have the capacity to improve survival when used appropriately [1, 2, 4]. In cases where the aetiology of the cardiac arrest is unknown, good quality bystander CPR and utilisation of the AED are indicated as first measures. This is especially important when transport times to hospital might be longer in rural or distant locations. This case was located in a rural golf club, with a public AED and educational guidance for club members. Furthermore, non-ST elevation myocardial infarction (NSTEMI) or other cardiac causes of cardiac arrest may not necessarily be ruled out with prehospital ECG, since ST segment elevation or depression is not present. Although no acute changes were seen on the 12-lead ECG in this case, performing one is important in order to detect any abnormalities, since these may influence the treatment and outcomes of patients [5].

Bystander CPR has previously been reported on a golf course following spontaneous collapse, albeit with a more straightforward cardiac aetiology [6]. Early CPR and utilisation of an AED are usually indicated for spontaneous cardiac arrest regardless of aetiology and were used in an appropriately early manner in this case. This case had a trickier differential diagnosis, with the possibility of intracranial and cardiac pathology, but the same early course of action was indicated. The quick and decisive decision-making of the prehospital staff ensured a rapid and appropriate transfer to a facility with all of the specialist resources required for the varied differential diagnoses, even though this was not the nearest hospital. A potential case might be made for prehospital personnel to convey casualties following cardiac arrest to nominated “cardiac arrest” centres in the same way that there are stroke and trauma centres. These would contain interventional cardiology, stroke, and neurosurgery services so that any potential aetiological factor might be addressed appropriately. In this case, the Major Trauma Centre was able to provide all of these services.

There is a wide differential diagnosis for pupil asymmetry, and it does not always indicate neurological pathology [7]. In this case, the anisocoria was irrelevant to the diagnosis, but, in an emergency situation, with limited history and requirement for urgent transfer and resuscitation, it has the potential to add uncertainty to the diagnosis and appropriate treatment strategy. An awareness of this phenomenon is important amongst prehospital and emergency practitioners, and the requirement for a focused, accurate, and timely history is paramount.
